# 2D Materials Enabled Next‐Generation Integrated Optoelectronics: from Fabrication to Applications

**DOI:** 10.1002/advs.202003834

**Published:** 2021-03-15

**Authors:** Zhao Cheng, Rui Cao, Kangkang Wei, Yuhan Yao, Xinyu Liu, Jianlong Kang, Jianji Dong, Zhe Shi, Han Zhang, Xinliang Zhang

**Affiliations:** ^1^ Wuhan National Laboratory for Optoelectronics Huazhong University of Science and Technology Wuhan 430074 P. R. China; ^2^ Institute of Microscale Optoelectronics Collaborative Innovation Centre for Optoelectronic Science & Technology Key Laboratory of Optoelectronic Devices and Systems of Ministry of Education and Guangdong Province College of Physics and Optoelectronic Engineering Shenzhen Key Laboratory of Micro‐Nano Photonic Information Technology Guangdong Laboratory of Artificial Intelligence and Digital Economy (SZ) Shenzhen University Shenzhen 518060 P. R. China

**Keywords:** 2D materials, modulator, photodetector, semiconductor laser, silicon photonics

## Abstract

2D materials, such as graphene, black phosphorous and transition metal dichalcogenides, have gained persistent attention in the past few years thanks to their unique properties for optoelectronics. More importantly, introducing 2D materials into silicon photonic devices will greatly promote the performance of optoelectronic devices, including improvement of response speed, reduction of energy consumption, and simplification of fabrication process. Moreover, 2D materials meet the requirements of complementary metal‐oxide‐semiconductor compatible silicon photonic manufacturing. A comprehensive overview and evaluation of state‐of‐the‐art 2D photonic integrated devices for telecommunication applications is provided, including light sources, optical modulators, and photodetectors. Optimized by unique structures such as photonic crystal waveguide, slot waveguide, and microring resonator, these 2D material‐based photonic devices can be further improved in light‐matter interactions, providing a powerful design for silicon photonic integrated circuits.

## Graphical abstract

2D materials have gained persistent attention in the past few years and they meet the requirements of complementary metal‐oxide‐semiconductor compatible silicon photonic manufacturing. A comprehensive overview of fabrication and evaluation of state‐of‐the‐art 2D photonic integrated devices for telecommunication applications is provided, including light sources, optical modulators, and photodetectors in on‐chip devices.

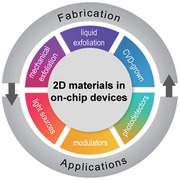

## 1 Introduction

With the unprecedented growth of data capacity, the demand of information transmission deployed in high‐speed transceivers is forcing communication devices to be employed with the fastest and most efficient components on the integrated platform.^[^ ^1^, ^2^, ^3^ ^]^ Silicon photonics, possessing low cost and high compatibility with complementary metal‐oxide‐semiconductor (CMOS) industry, is a promising solution in future large scale silicon photonic integrated circuits (PICs), bridging the information between the electrical domain and optical domain.^[^ ^1^ ^]^ However, traditional silicon PICs are difficult to efficiently generate, modulate, and detect light in a monolithic integrated silicon chip. Although the III–V materials can epitaxially grow on the silicon chip, they suffer from mismatch of lattice constant and thermal expansion coefficient in the interface between silicon and III‐V materials.^[^ ^4^, ^5^ ^]^ The defects in the interface also limit the optical and electrical performance of the ultimate devices.

Recently, 2D materials, such as graphene, black phosphorous (BP), transition metal dichalcogenides (TMDs) and so on, have been developed in optical communication,^[^ ^6^, ^7^ ^]^ biosensing,^[^ ^8^, ^9^ ^]^ biomedical,^[^ ^10^, ^11^ ^]^ laser sources,^[^ ^12^, ^13^ ^]^ and photodetectors.^[^ ^14^, ^15^ ^]^ Graphene has been widely investigated since it was exfoliated in the first time. It was utilized in high speed applications based on the high carrier mobility and was offered in photodetectors due to the zero bandgap. On the other hand, BP and TMDs have the intrinsic bandgaps exhibiting many‐body complexes which promotes a wider applications in numerous optoelectronic applications.^[^ ^16^ ^]^ Typically, they were demonstrated as hybrid structures to improve the performance of optoelectronic devices with their unique properties such as high carrier mobility and strong light‐matter interaction.^[^ ^17^, ^18^, ^19^, ^20^ ^]^ Besides, since the individual atomic planes are stacked by van der Waals force, 2D materials can be easily exfoliated from bulk materials and then transferred onto an integrated silicon chip without any adhesives.^[^ ^17^ ^]^ These merits allow 2D materials to be integrated in high speed silicon PICs and they have been overviewed in these applications, such as high‐efficiency light sources, high‐speed modulators and photodetectors especially in the telecommunication band of 1.55 µm.^[^ ^7^, ^18^, ^20^, ^21^, ^22^ ^]^

In these hybrid integrated silicon‐based optical devices with 2D materials, graphene takes the majority since it was first exfoliated in 2004.^[^ ^23^ ^]^ Over the past few years, 2D materials have been widely studied and have been continuously promoted.^[^ ^7^, ^18^, ^20^, ^21^, ^24^, ^25^, ^26^ ^]^ Monolayer graphene was found that it can absorb 2.3% of normal incident light,^[^ ^27^ ^]^ which is strong regarding the atom‐level thickness. However, this value is not sufficient for most practical applications due to the apparently weak light‐matter interaction in an all optical device. Therefore, transferring 2D materials on a silicon waveguide becomes an effective solution, where a long light‐matter interaction length generates along the waveguide. However, in order to enable 2D materials devices to obtain more advantages than traditional silicon platforms, it is still a critical issue to appropriately apply 2D materials on optical devices. Enhancing the light‐matter interaction with 2D materials is one of the most important issues we need to address. In order to increase the light‐matter interaction, several solutions, such as increasing the length, time and range of interaction, have been demonstrated in specific structures while maintaining a compact size. Specifically, the resonance in microring resonator (MRR) or micro‐disk is utilized to indirectly increase the length of interaction;^[^ ^28^, ^29^, ^30^, ^31^ ^]^ the slow light effect in photonic crystal waveguide (PhCW) is exploited in achieving large group index to extend the interaction time;^[^ ^32^, ^33^, ^34^ ^]^ the leaky‐mode in slot plasmonic waveguide will enhance the light‐matter interaction range.^[^ ^35^, ^36^, ^37^, ^38^ ^]^

In this review, we focus on the fabrications and applications of 2D materials in high speed optical communications including graphene, TMDs, MXene, and BP. We discussed recent applications employing waveguide‐based hybrid structures, such as strip waveguide, PhCW, MRR and plasmonic waveguide. One of the main topics we address in this review is to overview the integrated applications of 2D materials including fabrication methods, mechanisms of each functional devices and listing of state‐of‐the‐art 2D materials hybrid applications. We believe that this review will bring us a new perspective on 2D materials, and it enables an interdisciplinary research between 2D materials and optical communications.

## 2 Fabrication Methods for Integrated Devices Hybrid with 2D Materials

Since the first graphene sample exfoliated from graphite demonstrated outstanding properties,^[^ ^23^ ^]^ 2D material significantly draws researchers’ attention for its potential applications in many fields.^[^ ^19^ ^]^ A lot of efforts have been paid on the synthesizing, transferring and processing of 2D materials. Up to now, there are more than one method for synthesizing single‐layer or few‐layer 2D materials. The transferring and processing methods are also diverse. Since each method has its own strengths and weaknesses, we need to choose the most suitable type for the specific device. The transferring and processing method will add materials to the expected locations and remove the excess parts, which will affect the performance of the optical device eventually.

There are several methods for preparing 2D materials. To apply 2D materials in real applications, not only the large scale is considered, but also the quality is paid special attention. In this section, the fabrication methods of integrated devices will be briefly reviewed.

### 2.1 Fabrication Methods for Devices Integrated with Mechanical Exfoliated 2D Materials

Each layer in a 2D material consist of dangling‐bond‐free lattice, and is weakly bound to its neighboring lattice via van der Waals interaction. This enables the isolation of the atomic layers from naturally occurring crystals or artificially grown bulk materials by simply mechanical exfoliation. As a typical approach to yield 2D materials, it is feasible to access the highest quality with lower defect density, higher crystallinity, and less impurity contamination.^[^ ^39^ ^]^ However, the mechanical exfoliation process is a time‐consuming task due to the random natures of flake size, thickness, and quality. This would induce the scale challenge due to the repetition of manual labor. To address this issue, a robotic system, which can identify, analyze, operate the exfoliated 2D flakes has been developed.^[^ ^40^ ^]^ This system involves the deep learning to study 2D flake morphology, largely reducing the manual effort for scale production.

**Figure 1:**
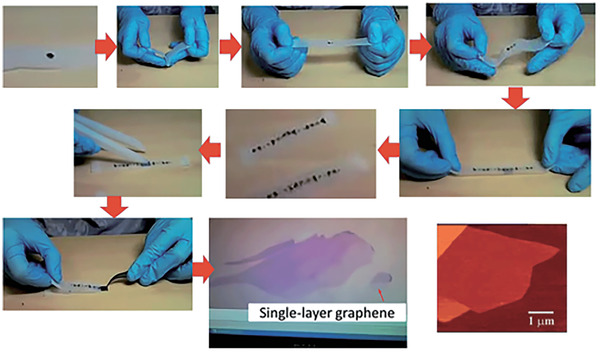
Illustrations of the mechanical exfoliation methods. Mechanical exfoliation method by repeated pealing of highly ordered pyrolytic graphite. Reproduced with permission.^[^ ^42^ ^]^ Copyright 2015, The Royal Society of Chemistry.

As the most well‐known 2D material, graphene is an amazing scientific finding in 2004 by Novoselov and Geim.^[^ ^23^ ^]^ Described in their original paper, the graphene is prepared by mechanical exfoliation (repeated peeling) of graphite using the commonly available scotch‐tape. Few layers and single layer of graphene are found to attach the substrate by van der Waals forces in free‐standing form.^[^ ^41^ ^]^ To isolate graphene from adjacent graphite layers, a stick tape can be utilized to peel off graphite flakes gradually and repeatedly, as shown in **Figure** **1**.^[^ ^42^ ^]^ A neat silica substrate is usually employed for receiving graphene by re‐building the van der Waals bondings between graphene and the substrate. Despite being only one atom thick, graphene is reported to absorb a significant (2.3%) fraction of incident white light, as intriguingly related to the fine‐structure constant.^[^ ^27^ ^]^ The optical visibility of transferred graphene on silica is further enhanced by interference colors, making graphene directly visible by the human eye. The combined breakthroughs of the Scotch‐tape method make it outstanding in plenty of reports prior to 2005, where even monolayer graphene was observed and lead to a Nobel prize in 2010. Since then, the easy‐to‐access graphene has entered the gate of scientific researches. Up to now, graphene can be exfoliated in large‐area by layer‐engineered exfoliation (LEE).^[^ ^43^ ^]^ Different metals are utilized to peel off selective layer of graphene thanks to the different interfacial binding energy. Metal Ni can be deposited onto the entire WS_2_ layers to exfoliate a continuous WS_2_ monolayer.^[^ ^44^ ^]^ Similarly, TMDs and hexagonal boron nitride (hBN) can be mechanically exfoliated in large scale by using an atomically flat gold tape.^[^ ^45^ ^]^ Huang et al. demonstrated a universal mechanical exfoliation method with the help of gold.^[^ ^46^ ^]^ The bulk material on the tape is brought in contact with the gold layer and the major portion of the material is peeled off by the adhesive tape. They tried 40 types of 2D monolayers by this method in their demonstrations.

Exfoliated 2D materials can be directly transferred onto the target substrate to form the desired devices when deposited with metal contact, such as transistors^[^ ^47^, ^48^ ^]^ and photodetectors.^[^ ^49^, ^50^, ^51^ ^]^ Even though exfoliation method can obtain 2D materials with excellent quality, how to prepare large‐scale and uniform 2D materials in the industrial process is still an urgent problem to be solved.

### 2.2 Fabrication Methods for Devices Integrated with Liquid Exfoliated 2D Materials

Liquid exfoliation can be used for mass production of nanosheets. Nanosheet is directly produced in liquid phase, so it has inherent machinability, and can be easily form composite materials, coatings or films, which is conducive to its use in various applications.^[^ ^52^, ^53^ ^]^ In addition, size control can be achieved by centrifugation, so that application and basic research can be processed. Protecting the nanosheets with appropriate solvents will allow large‐scale production under environmental conditions, greatly simplifying the future development of the material.^[^ ^54^ ^]^

**Figure 2:**
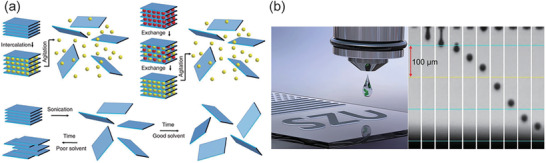
Fabrication process for devices integrated with liquid exfoliated 2D materials. a) Schematic description of the three liquid exfoliation mechanisms by ion intercalation (top‐left), ion exchange (top‐right) and sonication‐assisted exfoliation (bottom). b) Schematic of inkjet printing (left); stroboscopic camera view of printing droplet sequences (right). a) Reproduced with permission.^[^ ^57^ ^]^ Copyright 2013, American Association for the Advancement of Science. b) Reproduced with permission.^[^ ^78^ ^]^ Copyright 2019, Springer Nature.

In 2011, TMDs was synthesized by liquid exfoliation from layered materials.^[^ ^55^ ^]^ In 2014, few‐layers BP was exfoliated from bulk material and successfully applied in field‐effect transistors.^[^ ^56^ ^]^ The liquid exfoliation method became popular in obtaining 2D materials, as detailly illustrated in **Figure** **2**a.^[^ ^57^ ^]^ To exfoliate layer from bulk material, we need to weaken the interlayer attraction. Ions in the liquid can be absorbed into the spacing between layers and then the layers can be separated by agitation (such as shear, ultrasonication, or thermal),^[^ ^58^ ^]^ shown as top‐left of Figure 2a. In some metal oxides materials, there are ions between the layers to balance the surface charge.^[^ ^59^ ^]^ In the liquid environment, ion exchange method can replace the original surface ions to liquid ones, then an agitation will result in exfoliation dispersion, as shown in top‐right of Figure 2a. Sonication‐assisted exfoliation exposes the layered material to ultrasonic waves in a solvent.^[^ ^60^ ^]^ High‐energy jets in the bubbles generated from ultrasonic will tear up the layered crystallites and produce exfoliated nanosheets, as shown in bottom of Figure 2a. Since liquid exfoliation methods are of significant convenience in synthesizing, it have become the main option of preparing a large amount of 2D material in target devices.

Progressed from the graphics and newspaper industry, inkjet printing is a cutting‐edge method for efficiently transferring 2D materials on small‐footprint integrated photonic devices, which has the advantages of low cost, good controllability, and scalability.^[^ ^61^, ^62^, ^63^, ^64^ ^]^ 2D materials including graphene,^[^ ^65^, ^66^, ^67^, ^68^, ^69^, ^70^ ^]^ TMDs,^[^ ^71^, ^72^ ^]^ boron nitride,^[^ ^73^ ^]^ BP ^[^ ^74^ ^]^ can be produced as liquid dispersed nanosheets via liquid phase exfoliation from layered crystals. This is ideally suitable for producing printable inks. An appropriate choice of ink solution is critical. High viscosity solution like N‐methyl‐2‐pyrrolidone (NMP) and dimethylformamide (DMF) are commonly used for ink formulation since they are widely used in the liquid exfoliation processes for 2D materials.^[^ ^75^ ^]^ Li et al. proposed a universal approach to integrate 2D crystals inks with high efficiency, which is mainly based on a distillation‐assisted solvent exchange method combining with polymer stabilization.^[^ ^71^, ^76^ ^]^ This enabled them to formulate stable, high‐concentration, environmentally friendly and printable inks for graphene and MoS_2_, allowing for accurate jetting and printing resolution of ≈80 µm on silicon‐on‐insulator (SOI) substrate. However, the common solvents (e.g., NMP, DMF) used for 2D material‐based inks are toxic and require annealing during printing or in post‐processing for solvent removal. As a result, this method will impair the performance of 2D crystals even if it can remove the polymers.^[^ ^72^, ^77^ ^]^ Recently, MXene nanosheet is inkjet printed on silica glass, SOI wafer, side‐polished fiber, polyethylene terephthalate (PET) film and gold mirror substrate as shown in Figure 2b.^[^ ^78^ ^]^ The metallic titanium carbide Ti_3_C_2_T_x_ nanosheets ink formulated by isopropanol (IPA) is well‐controlled, non‐toxic and binder‐free. Different patterns such as dots matrix and stripes can be printed by changing the distance of the inter‐droplet distance. Especially, inkjet printing could also be used to create heterostructures of the various 2D materials on demand.^[^ ^77^, ^79^, ^80^, ^81^, ^82^ ^]^ The appeal of this technology lies in it being a non‐contact, material‐conserving, additive patterning and mask‐less approach with potential for large‐scale fabrication of 2D material‐based devices. However, there are challenges in several aspects, such as patterning dimensions, clogging of nozzle, low concentration of solution, jetting accuracy and reliability and so on, before inkjet printing can be adopted in real industrial applications.

### 2.3 Fabrication Methods for Devices Integrated with CVD‐Grown 2D Materials

Since the exfoliation method is limited in large scale production, a chemical vapor deposition (CVD) method is involved to obtain large scale 2D materials. Li et al. transferred large‐area graphene films to a substrate while maintaining the high electrical conductivity and high optical transmittance.^[^ ^83^ ^]^ Graphene grown on Cu ^[^ ^84^ ^]^ (or Ni ^[^ ^85^ ^]^ etc.) by CVD process possesses a large‐area and high mobility albeit not as much as mechanical exfoliation method. In CVD method, graphene is grown by using a mixture of methane and hydrogen at temperatures up to 1000 ℃ in the furnace. By placing the copper foil above the oxide substrate and leaving a gap between them, the speed of graphene growth by CVD was promoted significantly.^[^ ^86^ ^]^ Since the air flow can be adjusted, heterostructures grown in CVD process were demonstrated in TMDs.^[^ ^87^ ^]^ Moreover, graphene can be directly grown in photonic crystal fiber by CVD method, and the length can reach half a meter.^[^ ^88^ ^]^ hBN single crystals was grown in 100 cm^2^ on a low‐symmetry copper vicinal surface by CVD method.^[^ ^89^ ^]^ MoS_2_ flakes grown in stack multilayer showed the layer‐controllable in CVD process.^[^ ^90^ ^]^

**Figure 3:**
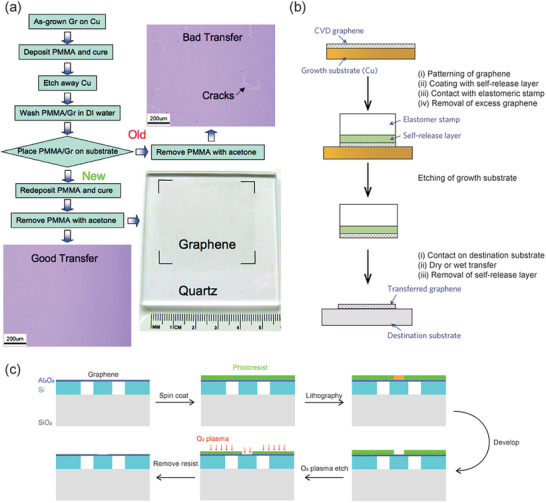
Fabrication process for devices integrated with CVD‐grown 2D materials. a) Modified process of graphene transferring (Gr = graphene). b) Schematic diagram of the self‐release layer method combined with a pick‐and‐place elastomer stamp. c) Schematic of the O_2_ plasma etch patterning method for graphene. a) Reproduced with permission.^[^ ^83^ ^]^ Copyright 2009, American Chemical Society. b) Reproduced with permission.^[^ ^98^ ^]^ Copyright 2013, Springer Nature.

Since graphene is the most widely applied material in devices, graphene grown by CVD method is need to be transferred for the next processing. To make sure graphene can be used on silicon‐based devices, it is necessary to separate useless metal substrate by a wet‐chemistry method. First, a layer of polymer or photo‐resist is spin‐coated onto the CVD‐grown graphene on the metal substrate then the underlying Cu is etched by Fe^3+^ solution at room temperature. The graphene/polymer film floating on the solution should be washed by deionized (DI) water for a few minutes and then transferred to the target silicon/silica substrate. This is followed by drying either at room temperature or on a hotplate. Afterward, the polymer on the graphene is dissolved by acetone and cleaned by ethanol. To avoid cracks in the transferring process, in **Figure** **3**a,^[^ ^83^ ^]^ a repeated polymer coating step after drying process can improve the quality of graphene.

Besides, TMDs can also be prepared by wet‐chemistry method that is similar to the method used in graphene, where the growth substrate is removed in hot base (NaOH or KOH) solution bath.^[^ ^91^, ^92^ ^]^ PtSe_2_, as one of the family of TMDs, can be prepared with a process similar to graphene. PtSe_2_ grown in CVD process was on the silica/silicon substrate and then transferred onto the desired substrate through a similar wet‐transfer process.^[^ ^93^ ^]^ Poly (methyl methacrylate) (PMMA) is spin‐coated onto the as‐grown PtSe_2_ and the underlying SiO_2_ layer is removed by a wet‐etching process using NaOH solution. The PtSe_2_ with PMMA is cleaned in DI water, and then directly transferred onto the target substrate. After drying at room temperature or on a hotplate, the residual PMMA is removed by acetone. An improved method utilizing the hydrophilic/hydrophobic interface of the TMD/substrate has also been reported, where the large scale TMD film can be easily peeled off from the substrate by water invasion, and then transferred to arbitrary surface with the help of polystyrene or polydimethylsiloxane (PDMS).^[^ ^94^, ^95^, ^96^ ^]^

At the platform of CVD, graphene and TMDs films can be grown in large scales,^[^ ^97^ ^]^ making the wet‐chemistry process works well in industrial manufacture. Besides, in order to transfer graphene on almost all surfaces, a PDMS stamp can be introduced to assist the process, which was successfully transferred in fragile polymer thin films and hydrophobic surfaces.^[^ ^98^ ^]^ Graphene patterned by oxygen plasma will be spin‐coated with a self‐release layer and then contact with the PDMS stamp, which will be dry/wet transferred to the destination substrate. With this method, graphene can be patterned before it gets transferred, as detailly illustrated in Figure 3b.

After the CVD transferring, in most cases, the chip will be covered with a large area of 2D material. However, only the core part we want to coat with 2D material, and the extra part should be removed in case of increasing unnecessary losses in the final optoelectronic devices. In order to get the specific pattern of the graphene, O_2_ plasma etching brings a quick and direct way. Multiple graphene layers can be sufficiently etched away in less than several seconds even if in large area for industrial usage,^[^ ^99^ ^]^ which has been widely used in the manufacture of graphene and other 2D materials devices. In this method, as shown in Figure 3c, the sample to which graphene has been transferred is spin‐coated first, followed by electron beam lithography (EBL) or ultraviolet (UV) lithography, and then a short oxygen plasma step is used to transfer the photoresist pattern onto the graphene sheet. Photoresist plays as a mask to protect the core part of graphene in this process. Finally, after cleaning the photoresist, we can get the desired graphene pattern on the chip.

Moreover, graphene can be carefully etched by singular sheet etching process when it is exfoliated from a bulk graphite.^[^ ^100^ ^]^ Exfoliated MoS_2_ nanosheets can be thinned through a thermal annealing process.^[^ ^101^ ^]^ BP can be encapsulated by graphene and get pattern etched directly through EBL.^[^ ^102^ ^]^ Those methods allow 2D materials to be processed and manufactured more precisely.

## 3 Light Emission Devices

Since the quantum confinement effect, 2D materials exhibit attractive optical properties, including tunable bandgap, spin–valley correlation, and large exciton binding energy, which enable 2D materials to be a promising candidate for light emission device applications. In this regard, we summarize the recent progress of 2D material‐based light emission devices in this section, including laser source and single photon source, in particular, TMDs, perovskites, and hBN.

### 3.1 Laser Source

**Figure 4:**
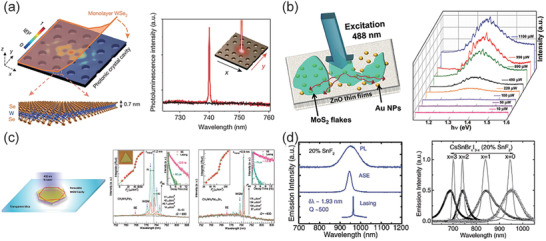
Laser sources with 2D materials enhanced. a) Schematic of WSe_2_‐Photonic crystal cavity nanolaser with its PL spectrum in the right. b) Schematic of MoS_2_‐Au‐ZnO NIR random laser with the PL measurement of samples. c) Lasing characterizations of perovskites whispering‐gallery‐mode nanocavities. d) NIR laser and ASE characterizations of tin‐based perovskites. a) Reproduced with permission.^[^ ^22^ ^]^ Copyright 2015, Springer Nature. b) Reproduced with permission.^[^ ^105^ ^]^ Copyright 2018, American Chemical Society. c) Reproduced with permission.^[^ ^106^ ^]^ Copyright 2014, American Chemical Society. d) Reproduced with permission.^[^ ^107^ ^]^ Copyright 2016, WILEY‐VCH GmbH.

Since the first ruby laser was demonstrated in 1960, it has been considered to be one of the greatest inventions in the 20th century owing to its wide applications. As the rapid development of semiconductor industry and urgent demand of high‐performance laser source for next generation optoelectronic devices, the 2D material‐based integrated laser source has become a worldwide investigated hot spot. Motived by this point, intensive investigations have been carried out. The first 2D material‐based nanoscale laser source was demonstrated by Wu et al.^[^ ^22^ ^]^ By employing high gain monolayer WSe_2_ and a photonic crystal cavity, an ultralow threshold of nanowatt laser source was achieved, as shown in **Figure** **4**a. Moreover, a Q‐factor of 10^4^ was obtained, which was beneficial for the realization of lasing. Subsequently, various TMDs were employed to fabricate 2D material‐based laser devices, including monolayer and multilayer MoTe_2_^[^ ^103^ ^]^ and MoS_2_/WSe_2_ heterostructure.^[^ ^104^ ^]^ In sharp contrast to the first demonstration of 2D material‐based nanoscale laser source, the above mentioned 2D material‐based laser were operated at room temperature, which is more suitable for practical application. Moreover, integrated with silicon (Si), the laser devices exhibited higher Q‐factor. Noticeably, the photo energy of the incident light below the bandgap of Si could be absorbed. Therefore, the bandgap of Si is critical since the spontaneous emission energy of the gain medium cannot be larger than it.

Random laser is different from traditional laser which has a cavity to amplify the light, but by disorder‐induced scattering process. Due to its ease of fabrication and high on‐chip integration, random laser has been employed in a variety of applications. However, most investigations are explored in visible band. Recently, Dixit et al. exhibited a near‐infrared (NIR) random laser based on multilayer MoS_2_, as shown in Figure 4b.^[^ ^105^ ^]^ The power dependent photoluminescence (PL) spectrum was measured to calculate the threshold of lasing process, and to get an insight of charge transfer mechanism in the MoS_2_/Au nanoparticles/ZnO heterostructures. The author inserts Au nanoparticles between MoS_2_ and ZnO thin flakes, which can enhance the light scattering due to the surface plasmon resonance process. It can be observed that the sample used to generate the random laser has a clear PL spectrum in the NIR band. Noticeably, the emission intensity increased abruptly when the incident laser power is beyond 490 µW. The lasing intensity was nonlinear with laser power, and a clear inflection point can be found at ≈500 µW. The generation of random laser can be attributed to three aspects: first, the significant enhanced multiple scattering caused by Au/ZnO disordered structure; second, the surface plasmon resonance process caused by the inserted Au nanoparticles; third, enhanced charge transfer from ZnO to thick MoS_2_ flakes. These interesting findings provide new opportunities to achieve on‐chip integrated NIR random laser.

Although nanolasers based on III−V nanowire lasers have made significant progress at room‐temperature, the problems of high Auger losses and low quantum efficiencies remain to be resolved. The organic‐inorganic lead halide perovskites nanoplatelets have attracted considerable attention due to the excellent efficiency in solar‐cell application. Furthermore, a recent investigation proved these perovskites thin films have excellent optical gain properties in a wide wavelength range. Zhang et al. exhibited a study of NIR nanolasers based on organic–inorganic perovskite operating at room‐temperature.^[^ ^106^ ^]^ By introducing a whispering gallery mode cavity, they achieved NIR laser with wide mode‐tunability and low thresholds. Figure 4c left shows the schematic diagram of the device. The Q‐factor increased linearly with the edge length, and the perovskite can compensate the scattering and radiation loss in the cavity. Figure 4c mid and right present the power‐dependent emission spectra of CH_3_NH_3_PbI_3_ and CH_3_NH_3_PbI_3_‐aBra, respectively. As the pump laser increased, the lasing action occurred. To confirm the potential of on‐chip integrated optoelectronic devices, the author demonstrated the lasing onto various conductive bases including Si, Au, and indium tin oxide (ITO) with the same size of CH_3_NH_3_PbI_3_ sample. Lasing behavior is achieved for all the conductive bases used in the experiment at room temperature. The threshold of laser is the same for these conductive bases, which is due to the exciton avalanche process occurred in a picosecond time‐scale, and it is much shorter than that of the carrier transfer/trapping at the surface/interfaces. These outcomes indicate organic‐inorganic lead halide perovskites nanoplates can act as a promising candidate for the realization of NIR solid state nanolasing.

Xing et al. demonstrated an investigation of solution‐processed tin‐based perovskite for NIR lasing.^[^ ^107^ ^]^ Although the result exhibited a poor performance in photovoltaic behavior, but the tin‐based halide perovskites (CsSnX_3_, X = Br, I) show an excellent optical gain properties in the NIR band up to 1 µm. Stimulated emission was achieved with a low‐threshold of 6 µJ cm^–2^ and a large gain of 200 cm^–1^ from 20% concentration SnF_2_ added into CsSnI_3_. Figure 4d shows the PL, ASE and single mode of the low‐threshold lasering of CsSnI_3_ contains 20% concentration SnF_2_. The FWHM of the laser is 1.9 nm, and the corresponding Q‐factor is 500. By changing the concentration of bromides and iodides, the ASE peak can be modified from 700 to 950 nm at room temperature. Unlike leaded halide perovskites, which are restricted to around 800 nm, these light emission properties of Sn‐based perovskites are comparable or even better than that of lead‐based perovskites.

Apart from infrared band, visible band also drawn considerable attention due to its various applications. Recently, the formation of MXene quantum dots, combined with the quantum confinement and edge effects, enable this attractive novel 2D material holds great potential for white laser source. Huang et al. demonstrated a white laser range from 490 to 613 nm based on V_2_C MXene quantum dots, who presented the laser linewidth of 490, 545, 587, and 613 nm.^[^ ^108^ ^]^ These outstanding findings indicate that MXene based quantum dots are promising for visible laser source applications. Additionally, mono‐ and few layer MoS_2_ is also employed to generate ultrafast visible laser pulse.^[^ ^109^ ^]^ By means of mode‐locked laser process, visible laser pulses (522, 607, and 639 nm) with picosecond pulse width and repetition rate larger than 100 million hertz were achieved. Furthermore, the layer numbers of MoS_2_ also presented a strong impact on the lasing process. As the thickness of MoS_2_ increased from monolayer to 3 layer, the average output power of obtained laser pulses was increased to 46 mW, which was much higher than that of monolayer condition. Meanwhile, the threshold was decreased to 0.71 W as well. MoS_2_ has the advantages of being broadband, ultrafast laser source.

### 3.2 Single Photon Source

**Figure 5:**
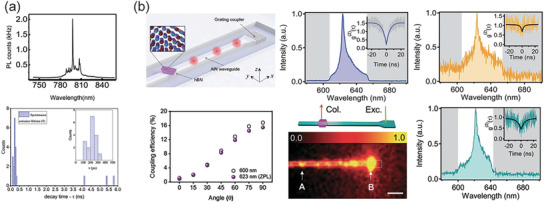
Performance of single photon sources. a) The measured PL intensity and histogram of spontaneous emission lifetimes. b) Schematic diagram of the hBN based waveguide device. (top‐left) PL spectrum of the device with and without modified collection technique, respectively. (top‐mid and top‐right) Coupling efficiency of the system as a function of the emitter's angle. (bottom‐left) Schematic diagram of excitation spot fixed at the grating coupler and the collection scanned across the sample area. (bottom‐mid) PL spectrum of the device with reverse collection technique. (bottom‐right) a) Reproduced with permission.^[^ ^111^ ^]^ Copyright 2018, American Chemical Society. b) Reproduced with permission.^[^ ^112^ ^]^ Copyright 2019, WILEY‐VCH GmbH.

The integration of on‐chip single photon sources plays a key role in the optical quantum information processing systems. Intensive investigations have been carried out to pursue highly efficient and stable single photon source. However, conventional single photon source, such as quantum dots, suffers from the weaker confinement and long spin coherence, which strictly restrict its further development. Fortunately, the 2D material‐based single photon source can overcome the issues mentioned above, and it is thought to be a promising approach to realize high performance single photon emitter. In this regard, TMDs have been employed to fabricate single photon emitters. He et al. demonstrated an investigation of monolayer WSe_2_ based single photon emitter. A series of sharp line width emissions on the lower energy side of the delocalized exciton emission were obtained. Photon antibunching from correlation measurements was carried out to characterize the single photon process.^[^ ^110^ ^]^ Metallic substrates can reflect nearly all of the photons toward the collecting objective. Additionally, metallic nanoparticles on a metal surface can be considered as native antennas. By taking these advantages, the spontaneous emission rate of atomic monolayers TMDCs can be increased significantly. Tripathi et al. recently demonstrated strain induced single photon sources by utilizing exfoliated monolayer WSe_2_ on a rough silver substrate coated by 3 nm Al_2_O_3_.^[^ ^111^ ^]^ Combined with simulated calculations, the outcomes represent a huge step for ultracompact integrated nanoplasmonic devices development. To have an insight of the spontaneous emission rate enhancement, time‐resolved spectra measurement of the fabricated device was carried out, as shown in **Figure** **5**a. The inset shows a partial of the full measure range, a 100 ps lifetime of the PL was recorded, which indicated enhancement behavior occurred through coupling to plasmonic modes in the quantum emitter.

To further testified the origin of the enhancement behavior caused by the coupling of quantum emitters to plasmonic resonance process in the silver surface, the spontaneous emission decay rate was calculated by finite‐difference time‐domain (FDTD) analysis. The calculation indicates that the enhancement of decay rate is caused by a nanocone antenna. Furthermore, the optimum coupling efficiency is around the edges of the cone.

Apart from TMDs, hBN was also employed for single photon emitter, which can operate at room temperature. Kim et al. presented a room temperature hBN based waveguide single photon emitter, as shown in top‐left of Figure 5b.^[^ ^112^ ^]^ The generated zero‐phonon line of the device was located at 623 nm and with a linewidth of 4.24 nm (top‐mid of Figure 5b), the inset of it indicated the zero‐delay time value of *g* ^(2)^(0) was 0.12, confirmed the single‐photon nature of the emitter. Moreover, with a modified collection technique, the zero‐delay time was efficiently enhanced, which was mainly due to the increased background than that of local collection configuration, as shown in top‐right of Figure 5b. To evaluate the coupling efficiency of the system, FDTD simulations were carried out. The calculated coupling efficiency presented a maximum value of 15.5% at 623 nm when the emission polarization was perpendicular to the waveguide direction, as shown in bottom‐left of Figure 5b. To further evaluate the performance of the system, reverse collection measurement was carried out. As shown in bottom‐mid and bottom‐right of Figure 5b, the excitation spot was set at the end point on the grating coupler and the collection scanned the waveguide across the sample area. The zero‐delay time was measured to be 0.42, which was higher than that of nonlocal measurement aforementioned. All these outcomes indicate that hBN was a promising material for being integrated on chip single photon quantum emitters.

## 4 2D Material‐Based Modulators

Optical modulators are ubiquitous components when transferring information from one media to another. Benefit from unique properties of 2D materials, optical modulator is developed with better performance in terms of faster response time, lower energy cost and sufficient light‐matter interactions. In this part, the state‐of‐the‐art optical modulators based on 2D materials will be listed, whose mechanisms are also summarized and discussed.

### 4.1 Mechanisms of 2D Modulators

In telecom systems, optical modulator converts the signal from electrical domain to optical domain, which is the first component in the optical signal processing chain. An idea optical modulator should have the merits of large bandwidth, low energy consumption and simple fabrication method, where 2D materials meet the demands of CMOS‐compatibility and high carrier mobility. In this section, we will focus on the mechanisms of modulators and then present the state‐of‐the‐art modulators based on 2D materials.

#### 4.1.1 Electro‐Optical Modulators with 2D Materials

As the bridge of electrical and optical signals, the electro‐optical (E/O) modulator is one of the most widely used modulators in communication systems. Integrated in PICs with additional electrical components, the E/O modulators play vital roles in the fields of optical interconnects, biosensing, quantum photonics technology, and so on. In future large‐scale PICs, power consumption, bandwidth and integration density are critical considerations in optoelectronics, and therefore new materials and novel structures for E/O modulators were proposed.

In the past decades, lithium niobate modulators have dominated commercial modulators for its fast Pockels‐effect. However, the weak light confinement due to low refractive index of lithium niobate strongly limits its integration density. III–V materials, such as InP and GaAs, are also promising materials with high refractive index and high Kerr coefficient. Unfortunately, they are not compatible with group‐IV semiconductor materials in the mature SOI platform. Silicon‐based modulators have been widely investigated since they are CMOS compatible. However, the silicon E/O modulator is not an integrated component with low energy consumption since it relies on plasma dispersion effect. As the scale of integration increases, energy consumption will bring us into an unbearable dilemma.

2D materials have attracted considerable attention for E/O modulation in recent years. Compared with traditional bulk materials, they exhibit unique electrical and optical properties. For instance, graphene, as the best‐known 2D material, exhibits ultra‐wide absorption band spectrum,^[^ ^113^ ^]^ tunable Fermi level,^[^ ^19^ ^]^ ultrahigh carrier mobility ^[^ ^114^ ^]^ as well as easy fabrication.^[^ ^115^ ^]^ Those unique properties will effectively reduce the energy consumption and increase the bandwidth of the E/O modulator. The high‐speed E/O modulator is always achieved with a capacitor structure where the 2D material serves as the electrode over a waveguide region. Take graphene as an instance, graphene absorbs the evanescent field of light propagating in the waveguide, thus modulates the intensity of signal light. High carrier mobility reduces the RC time constant in the specific design, thus the intrinsic speed of E/O modulators can be promoted. Kovacevic et al. illustrated the theory of a double‐layer graphene on top of a slot waveguide and it is potential to achieve response bandwidth over 100 GHz.^[^ ^116^ ^]^ The Fermi level of graphene is related to the applied bias and consequently affect the optical conductivity of graphene.^[^ ^117^ ^]^

#### 4.1.2 Thermo‐Optical Modulators with 2D Materials

Microheaters on silicon PICs are one of the fundamental tuning units to make the circuits reconfigurable and versatile. Microheaters normally rely on the thermo‐optical effect, which is defined as the change of the refractive index of optical material with the temperature. Thanks to the large thermo‐optical coefficient of ≈at 1.5 µm wavelength in silicon,^[^ ^118^ ^]^ microheaters on silicon photonic platforms have been extensively studied. Traditional microheaters in SOI structures mainly consist of metal electrode, SiO_2_ layer, silicon waveguide and silica substrate,^[^ ^119^, ^120^, ^121^ ^]^ where the metal electrode will be heated by a bias voltage. The SiO_2_ layer can prevent the metal electrode from absorbing light. However, due to the low thermal conductivity of SiO_2_ (1.44 W m^–1^ K^–1^),^[^ ^122^ ^]^ the SiO_2_ layer inevitably impedes heat transport and dissipation in the thermo‐optical modulators. As a result, the energy management for the metallic microheaters is facing critical challenges. Besides, the operation speed of the microheaters is rather slow due to the intrinsic slow thermal diffusivity. Thus, microheaters are often used in low speed on‐chip tuning units especially in optical switches and routers.^[^ ^123^, ^124^, ^125^ ^]^

In the past years, various excellent schemes, such as free‐standing racetrack resonators^[^ ^124^ ^]^ and optimized doping waveguides,^[^ ^126^ ^]^ have been demonstrated to improve the performances of metallic microheaters. However, none of them realized a stable, highly efficient and low energy consumption thermo‐optical modulator in the silicon platform. Thanks to the rapid growing of graphene applications, the excellent property of high intrinsic thermal conductivity (up to 5300 W m^–1^ K^–1^) gradually attracted attentions.^[^ ^127^ ^]^ Besides, the low light absorption (≈2.3%)^[^ ^27^ ^]^ for normal incidence in telecommunications band allows to greatly reduce light absorption in thermo‐optical modulators, thereby heralding as a promising candidate as transparent heaters. The ultrahigh carrier mobility of graphene prompts it to become a fast response speed conductor. Other 2D materials like BP also have a relatively high carrier mobility that can be implemented in high‐speed thermo‐optical modulators, but their bandgap will also cause greater losses than graphene due to the light absorption. Due to the flexible fabrication compatibility compared with conventional optical devices, graphene microheaters based on integrated photonic platforms have shown promising prospects in recent years.

#### 4.1.3 All‐Optical Modulators with 2D Materials

In large photonic circuits, it is highly desirable to avoid the signal conversion between optical domain and electronic domain. Therefore, all‐optical modulator, which can control the signal light by another probe light, achieves the pure optical communication in the circuit. Conventional all‐optical modulators based on bulk materials are always limited by either the response time due to the intrinsic low carrier mobility or power consumption of nonlinear Kerr effect. All‐optical modulators using 2D materials are typically realized by mechanisms of Pauli blocking,^[^ ^128^ ^]^ nonlinear Kerr effect,^[^ ^129^ ^]^ optical doping,^[^ ^130^ ^]^ and the thermo‐optic effect.^[^ ^30^, ^131^ ^]^ In this case, 2D materials act as the light‐matter interaction media, in which the signal light will be modulated by another beam with a lager photon energy. However, the Kerr effect is always achieved by high power light beam in the optical fiber to excite the nonlinear phase shift, and the optical doping happens in terahertz (THz) wave modulation based on the variation of electrical conductivity, neither of which is suitable for integrated circuits. Thus, Pauli blocking becomes popular in achieving all‐optical modulation. As a result, electrons are excited from the valence band into the conduction band, which shifts the absorption threshold of graphene to higher frequency then eventually increases the transmission of the signal light due to reduced absorption.^[^ ^128^ ^]^ Additionally, the all‐optical modulator can be realized indirectly through the thermo‐optic effect, where the pump light interacts with the 2D material generating a heat then causes a variation of the refractive index of waveguide.

Although 2D material‐based all‐optical modulators have been studied for several years, the majority of all‐optical modulators are implemented by optical fibers. So far, only a few of them have been used in the field of integrated circuits for high‐speed modulation. The hybrid structure of 2D materials and microfiber can be easily matched with a standard fiber‐optic system for in‐fiber operation but not suitable for future PICs. In order to get a fast response time and to avoid limited bandwidth, therefore, the research work where the 2D materials are combined with silicon platform is becoming more attractive.

### 4.2 High Speed Modulators with 2D Materials

Optical modulators are of significant importance in the PICs, whose performance always limits the 3 dB bandwidth of the whole circuit. In telecom applications, it is essential to focus on small footprint, fast response time, low energy consumption and insertion loss for modulators. Thanks to the ultrahigh carrier mobility in graphene,^[^ ^114^ ^]^ ultrahigh speed modulation have been deeply reconsidered when jointed with traditional optoelectronic devices. In 2011, Liu et al. integrated a monolayer graphene on a silicon waveguide and realized a 1 GHz graphene‐silicon hybrid broadband optical modulator.^[^ ^132^ ^]^ Since the monolayer of graphene has only an absorption of 2.3% of vertical incident light, it is genius to put the graphene parallel to light beam. Hereafter, they optimized the structure to a double‐layer graphene (graphene‐Al_2_O_3_‐graphene) obtaining a relative high modulation depth of 0.16 dB µm^−1^, but the bandwidth was still at 1 GHz.^[^ ^133^ ^]^ This concept was clarified by Koester et al. in numerical calculations, which showed that 3 dB bandwidths over 120 GHz (30 GHz) were achievable at near‐ (1.55 µm) and mid‐ (3.5 µm) infrared bands.^[^ ^134^ ^]^ Monolayer (graphene‐oxide‐silicon, GOS) and double‐layer (graphene‐oxide‐graphene, GOG) design established the basic structure in graphene‐silicon hybrid E/O modulators.

#### 4.2.1 Graphene‐Based Modulators

**Figure 6:**
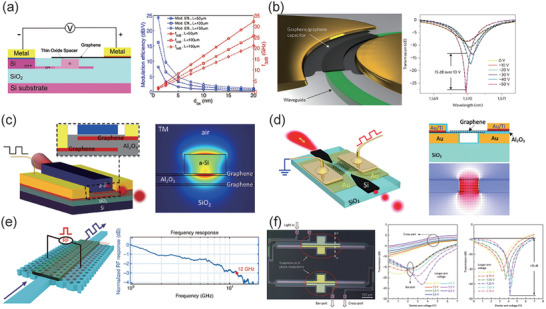
Graphene high speed E/O modulators. a) A 3D schematic drawing of the GOS type modulator, where the waveguide is doped to increase the bandwidth. The right figure describes the modulation efficiency and 3 dB bandwidth as functions of the thickness of the oxide spacer for different device length. b) Schematic of the high‐speed graphene‐MRR modulator on silicon nitride platform. (left) The transmission spectra for various applied bias voltages. (right) c) Schematic of the a‐Si modulator, where the two layers of graphene are separated by a Al_2_O_3_ dielectric on the bottom of an amorphous silicon waveguide. d) Schematic of an E/O modulator realized by graphene‐plasmonic slot waveguide. The injected light is guided and coupled from a taper silicon waveguide. e) Schematic of graphene‐PhCW hybrid E/O modulator with a 3 dB bandwidth of 12 GHz. f) Optical micrograph of the graphene‐MZI phase modulator. It shows the graphene works in absorption type (mid) and pure phase modulation type (right). a) Reproduced with permission.^[^ ^135^ ^]^ Copyright 2016, WILEY‐VCH GmbH. b) Reproduced with permission.^[^ ^28^ ^]^ Copyright 2015, Springer Nature. c) Reproduced with permission.^[^ ^137^ ^]^ Copyright 2016, American Chemical Society. d) Reproduced with permission.^[^ ^38^ ^]^ Copyright 2017, The Royal Society of Chemistry. e) Reproduced under the terms of Creative Commons Attribution 4.0 (CC BY).^[^ ^33^ ^]^ Copyright 2019, The Authors, published by De Gruyter. f) Reproduced with permission.^[^ ^138^ ^]^ Copyright 2017, Springer Nature.

The 2D material‐based high‐speed modulators were immersed in each unique structure in the past decade. The GOS structure used monolayer graphene on top of silicon benefits from simple fabrication process. Hu et al. improved the modulation efficiency to 1.5 dB V^−1^ and bandwidth to 5.9 GHz (with 10 Gb s^−1^ in experiment) by introducing a doped silicon waveguide in GOS structure, as shown in **Figure** **6**a.^[^ ^135^ ^]^ In addition to the GOS structure, the GOG structure has also been deeply investigated. Phare et al. demonstrated a 30 GHz bandwidth graphene electro‐optical modulator using MRR on silicon nitride platforms, as shown in Figure 6b.^[^ ^28^ ^]^ The MRR enhanced the light‐matter interactions in the GOG capacitor, resulting an impressive modulation depth up to 15 dB. Based on the resonance enhancement, Ding et al. demonstrated a graphene‐microring GOS modulator which shows a high modulation depth of 12.5 dB in the SOI platform.^[^ ^136^ ^]^ To avoid the influence of temperature changes on the refractive index, Dalir et al. buried the GOG structure under an amorphous silicon waveguide, and achieved a high modulation speed of 35 GHz. This modulator can keep its modulation speed in a wide range of temperatures, as shown in Figure 6c.^[^ ^137^ ^]^ Moreover, Ding et al. placed the GOG structure onto a metal plasmonic waveguide. This novel plasmonic slot waveguide structure can significantly reduce the insertion loss and the footprint, as shown in Figure 6d.^[^ ^38^ ^]^ To further increase the light‐graphene interaction time and keep a tiny footprint, Gan et al. used a monolayer graphene as an electrical gate on top of the photonic crystal nanocavity to control the Fermi level of graphene. They achieved a 10 dB modulation of cavity reflection for a swing voltage of only 1.5 V due to the resonance enhancement.^[^ ^47^ ^]^ Utilizing the slow‐light effect of PhCW, Cheng et al. designed a GOG‐PhCW modulator. In their design, a modulation depth of 0.5 dB µm^−1^ with a 3 dB bandwidth of 13.6 GHz was achieved, as shown in Figure 6e.^[^ ^33^ ^]^ However, the experiment showed a bandwidth of 12 GHz with modulation depth of 0.055 dB µm^−1^ due to the fabrication defects. Due to the special energy band diagram, the Fermi level of graphene can be controlled by external bias in the Pauli blocking regime, where only the real part of the effective index of graphene is varied. Thus, a phase modulator can also be realized by GOG/GOS structure. Sorianello et al. demonstrated a 5 GHz bandwidth GOS phase modulator in a MZI structure, as shown in Figure 6f.^[^ ^138^ ^]^

**Figure 7:**
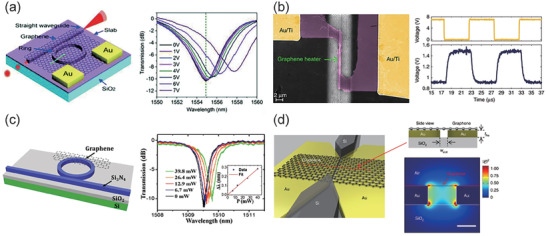
State‐of‐the‐art graphene thermo‐ and all‐ optical modulators. a) Highly efficient graphene microheater on a silicon MRR with a static thermo‐optic response of the MRR on the right. b) Photonic crystal waveguide was introduced to graphene thermo‐optical modulator based on slow light effect, where the slow light in PhCW significantly increased the length of light‐matter interaction. c) All‐optical modulator achieved by thermo‐optical effect in the transferred graphene thin layer based on a silicon nitride waveguide. The transmission spectra of MRR in relation to different power of pump light is illustrated on the right. d) Plasmonic waveguide all‐optical modulator with fast response time implemented by Pauli blocking in graphene. a) Reproduced with permission.^[^ ^140^ ^]^ Copyright 2015, The Royal Society of Chemistry. b) Reproduced with permission.^[^ ^32^ ^]^ Copyright 2017, Springer Nature. c) Reproduced with permission.^[^ ^30^ ^]^ Copyright 2017, Springer Nature. d) Reproduced with permission.^[^ ^37^ ^]^ Copyright 2019, Springer Nature.

With the development of 2D material‐based integrated devices, high‐speed modulators also achieve a fast response time in thermo‐ and all‐ optical modulators. Light absorption in graphene is much less than metal contact in thermo‐optical modulators. Therefore, it is useful to replace the metal heater with a graphene heater. Besides, since the graphene is transferred onto the chip surface, graphene heaters on the waveguide without the thick SiO_2_ isolation layer can further enhance the efficiency of heat conduction. As early as 2013, Kim and his colleagues developed a graphene microheater to implement a thermo‐optic mode extinction modulator in graphene plasmonic waveguide.^[^ ^139^ ^]^ The modulator consists of two crossed graphene microribbons and a polymer dielectric with high thermo‐optic coefficient. Once applying an external voltage, the long‐range surface plasmon polaritons mode transmitted in the long graphene strip can be cut‐off by the short graphene microheater with Joule heating. Using graphene as a microheater can greatly improve the efficiency of the thermo‐optical modulator. Later, it was further applied onto other optical resonance structures, and both the speed and efficiency were enhanced. Gan et al. proposed a highly efficient graphene microheater on a silicon MRR structure with a simple fabrication flow, as shown in **Figure** **7**a.^[^ ^140^ ^]^ The MRR on an SOI wafer is fully covered by a monolayer graphene heater. Joule heating is generated when external voltage is applied on the graphene through the metal electrode. Thanks to the ultrahigh carrier mobility and thermal conductivity of graphene, the device achieved an ultrafast response time of ≈700 ns. Yu et al. optimized the shape of graphene heater on a microdisk resonator, which improved the thermal tuning efficiency to 0.48 nm mW^−1^.^[^ ^29^ ^]^ In 2017, photonic crystal waveguide was introduced to graphene thermo‐optical modulator based on slow light effect, as shown in Figure 7b.^[^ ^32^ ^]^ A 1.07 nm mW^−1^ thermal tuning efficiency (figure of merit of 2.543 nW s) is achieved with a fast response time of 525 ns. It is noticeable that graphene can be used as heaters on any complex surfaces due to its unique property of flexibility. Thermo‐optical modulators aimed at high efficiency modulation in the conception. However, with the gradually improvement of fabrication methods, the modulation speed has been raised to nanosecond scale, making applications in high‐speed optical circuits possible.

Graphene also frequently showed in all‐optical integrated modulators by several special methods, such as free space illumination,^[^ ^141^, ^142^ ^]^ microring resonator assisted by thermo‐optical effect^[^ ^30^, ^131^, ^143^ ^]^ and saturable absorption on optical waveguides.^[^ ^37^, ^144^ ^]^ Inspired by the all‐optical modulator on the silicon photonic crystal cavity, where the graphene is covered on the whole substrate illuminated by 1064 nm laser beam from free space,^[^ ^145^ ^]^ several waveguide‐based works were demonstrated. Sun et al. employed dielectric‐loaded waveguide^[^ ^141^ ^]^ and plasmonic waveguide^[^ ^142^ ^]^ to implement the graphene all‐optical modulator in each waveguide structure. However, due to illumination from the free space, it is not an exquisite method exploited in integrated interconnections. Qiu et al. transferred a graphene thin layer onto the silicon nitride waveguide, covered a length of MRR of 43.4 µm, and achieved a switch time of 253 ns, as shown in Figure 7c.^[^ ^30^ ^]^ Thermo‐optical effect is dominant in their modulator, and a tuning efficiency of 0.0079 nm mW^−1^ was measured in relation to the light power. Besides, the saturable absorption method was demonstrated in an all‐optical modulator on a plasmonic waveguide with an ultrafast response time of 260 fs.^[^ ^37^ ^]^ Due to the Pauli blocking in graphene, the graphene becomes a transparent material with optical pulse excitation. Moreover, the plasmonic waveguide will enhance the light‐matter interactions in graphene and further reduce the device size, as shown in Figure 7d.^[^ ^37^ ^]^ The same method was investigated on a straight waveguide with a response time of 1.65 ps.^[^ ^144^ ^]^ With the development of graphene all‐optical modulators, saturable absorption is undoubtedly the most suitable method for all‐optical modulation. The fast response time caters for high speed optical communications, but the extinction ratio needs to be improved by further enhanced optical structures.

2D material‐based integrated modulators are demonstrated by various unique optical structures, which enhance the performances in applications. Strip waveguide is the most commonly used optical structure in optical building blocks. It is low loss, easily fabricated and can be composed in PICs without changing optical circuits. However, it cannot obtain a large modulation depth since there is neither resonance effect nor strong light‐matter interaction in it. Instead, MRR with a resonance structure can improve the modulation depth greatly, but the footprint of MRR is typically tens of square microns. Thus, it is not easy to be integrated in a compact size. To address this problem, PhCW and slot waveguide is specially designed and used. In PhCW, slow‐light effect will significantly enhance the light matter effect, and it also has a small size comparable with strip waveguide even through the fabrication process is relatively complicated. Slot waveguides are compact and moderately difficult to fabricate. Combining with metallic nanostructures, the plasmonic effect in the graphene and metal brings a strong light‐matter interaction. It improves the response time without sacrificing the modulation depth. To couple into silicon waveguide in PICs, a taper need to be designed in the interface of the two types of waveguide connections. Under specific requirements, the on‐chip 2D material‐based modulators should be chosen accordingly.

#### 4.2.2 Other 2D Material‐Based Modulators

**Figure 8:**
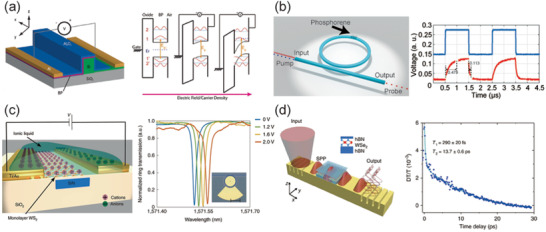
Designed and demonstrated modulators achieved by BP and TMDs. a) Schematic of BP based silicon waveguide mid‐infrared (MIR) electro‐optic modulator configuration. b) An all‐optical modulator based on BP demonstrated on a silicon MRR utilizing thermal‐optical effect with a temporal response signal. c) Schematic of the composite photonic platform of TMD monolayer (WS_2_ or MoS_2_) integrated with Si_3_N_4_ waveguide. d) Demonstrated ultra‐fast plasmonic modulator with WSe_2_ integrated on a metallic waveguide. a) Reproduced with permission.^[^ ^146^ ^]^ Copyright 2016, American Chemical Society. b) Reproduced under the terms of Creative Commons Attribution 4.0 (CC BY).^[^ ^131^ ^]^ Copyright 2020, The Authors, published by De Gruyter. c) Reproduced with permission.^[^ ^152^ ^]^ Copyright 2020, Springer Nature. d) Reproduced with permission.^[^ ^161^ ^]^ Copyright 2019, Springer Nature.

Integrated optical modulators based on BP, TMDs and other 2D materials have also been extensively studied with their unique properties different from graphene. In 2016, Lin et al. calculated the out‐of‐plane electric field controlled shift in BP's absorption edge, and proposed a BP based silicon waveguide mid‐infrared (MIR) electro‐optic modulator configuration, as shown in **Figure** **8**a.^[^ ^146^ ^]^ When BP film was in the quantum‐confined Franz‐Keldysh (QCFK) regime, the maximal attainable absorption and power efficiency was improved compared with graphene. Deng and his colleagues demonstrated the bandgap tuning in BP films with different thicknesses. By using a moderate displacement field up to 1.1 V nm^−1^ onto a 10 nm thick BP, a continuous bandgap tuning from ≈300 meV to below 50 meV was realized.^[^ ^147^ ^]^ In addition, electrically tuning the transmittance of BP in the infrared (IR) and MIR spectral ranges have also been successfully demonstrated, when using the BP/substrate configuration and illuminating from free space.^[^ ^148^, ^149^ ^]^ In 2019, Huang et al. demonstrated, for the first time, a waveguide integrated BP electro‐optic modulator for the MIR spectrum at wavelength from 3.85 to 4.1 µm. A modulation depth of ≈5 dB was achieved with a gate bias of −4 V, the maximum 3 dB bandwidth was measured to be 400 kHz, and the active footprint was only 225 µm^2^.^[^ ^150^ ^]^ Recently, an all‐optical modulator based on BP has also been demonstrated on a silicon MRR,^[^ ^131^ ^]^ as shown in Figure 8b. The BP film absorbs the pump light and causes an increase in temperature. Thus, the silicon waveguide is heated and the probe light is modulated. A tuning efficiency of 0.164 *π* mW^−1^ and a 3 dB bandwidth of 2.5 MHz are achieved. An alloy of BP, called black arsenic‐phosphorus (b‐AsP), has also been applied in optical modulator application. Liu et al. reported a b‐AsP based microheater on the silicon waveguide, achieving a high tuning efficiency of 0.74 nm mW^−1^ and a 10–90% rise/decay time of 30/20 µs.^[^ ^151^ ^]^

TMDs have also been explored in the application of optical modulation with different mechanisms. In 2015, Seyler et al. demonstrated electrically control of second‐harmonic generation (SHG) in WSe_2_ monolayer in a field‐effect transistor configuration,^[^ ^48^ ^]^ which revealed that electrically adjusting the optical characteristics of TMDs is a potential method for optical modulation. Recently, Datta et al. reported the composite photonic platform of TMD monolayer (WS_2_ or MoS_2_) integrated with Si_3_N_4_ waveguide, as shown in Figure 8c.^[^ ^152^ ^]^ Using electrostatically doping‐induced strong electrorefractive response in monolayer TMDs, a low‐loss modulator at near‐infrared was demonstrated, which benefits from the large bandgap and low optical absorption at telecommunication wavelengths of the monolayer TMDs. The device achieves an impressive Δ*n*/Δ*k* of ≈125, a *V*_*π*_ *L* of 0.8 V cm and a 3 dB bandwidth of 0.3 GHz. The TMDs based all‐optical modulators that utilize pump light to control the transmission of THz waves were demonstrated at first, with structures of MoS_2_‐on‐silicon,^[^ ^153^, ^154^ ^]^ MoS_2_‐on‐metaphotonic device,^[^ ^155^ ^]^ WS_2_‐on‐silicon,^[^ ^156^ ^]^ WSe_2_‐on‐silicon,^[^ ^157^ ^]^ and WSe_2_‐on‐metamaterial,^[^ ^158^ ^]^ etc. In ref. ^[^ ^156^ ^]^ the pump light caused the separation of electron‐hole pairs at the interface between WS_2_ and Si, thereby changing the free carrier absorption of the THz wave. Compared with the schemes that illuminated from free space, the integration of TMDs on waveguide undoubtedly increases the light‐matter interaction length greatly. Chen et al. demonstrated enhanced SHG with a structure of MoSe_2_ on silicon waveguide compared with free‐space SHG from TMDs.^[^ ^159^ ^]^ In 2017, Yang et al. reported all‐optical modulator with a WS_2_ on Si_3_N_4_ waveguide in the visible range.^[^ ^160^ ^]^ The 532 nm pump light is transmitted in the waveguide associated with the 640 nm probe light. Due to the photoluminescence (PL) effect of WS_2_, the 532 nm light will modulate and amplify the optical signal at 640 nm. This device achieves an extinction ratio of more than 10 dB. Recently, Klein et al. demonstrated an ultra‐fast plasmonic modulator with WSe_2_ integrated on a metallic waveguide.^[^ ^161^ ^]^ As shown in Figure 8d, utilizing the strong interaction between the surface plasmon polaritons (SPPs) and excitons in WSe_2_, the pump light from free space can cause a 73% variation in transmission of the probe light, with a response time of only 290 fs.^[^ ^161^ ^]^ Similar to BP,^[^ ^131^ ^]^ Wei et al. also demonstrated a PtSe_2_ based all‐optical silicon MRR modulator with 980 nm pump light illuminated from free space, achieving a tuning efficiency of 0.004 nm mW^−1^ and a 3 dB bandwidth of 2.4 kHz.^[^ ^162^ ^]^

## 5 2D Material‐Based Photodetectors

Integrated photodetectors have been raised to daily work with the rapid development of silicon photonics. The huge capacity of information transportation is urging us to develop ultra‐fast, low‐cost, large‐scale productive and integrated photodetectors. Graphene, with a tunable bandgap and ultra‐high carrier mobility, becomes the spotlight working in high speed photodetectors. Black phosphorus, with a direct bandgap from 0.3 (bulk) to 1.5 eV (monolayer) and high electron mobility, proved to be a low dark current and wide band detection material in photodetectors. Moreover, thanks to the nonzero bandgap in TMDs, they exhibit remarkably strong light absorption in the wavelength range from visible to IR and nonzero bandgap. TMDs can significantly increase the sensitivity of the detection even at zero gate voltage. Meanwhile, 2D materials can be grown with very low cost and can be transferred and patterned onto the silicon‐based optical devices, which gives the chance to be applied in the industrial manufacturing.

### 5.1 Mechanisms of 2D Photodetector

In the practical applications, photodetectors with 2D materials always have more than one generated photocurrent in a photodetector. The basic principle of photodetection is converting the intensity of light to the magnitude of current, which relies on injecting photons then generating photocarriers.^[^ ^26^ ^]^ Therefore, we briefly introduce the most common mechanisms in achieving photodetectors,^[^ ^163^ ^]^ including photovoltaic effect (PVE), photo‐thermoelectric effect (PTE) and photo‐bolometric effect (PBE).

PVE is the most common way to generate photocurrent. Under illumination, the Fermi energy of 2D materials is raised above conduction band and the current produced in bias voltage either by built‐in electric fields or external ones.^[^ ^164^ ^]^ The separated electron‐hole (e‐h) pairs excited by photoluminescence will fill in the PN junctions created by positive (p‐type) or negative (n‐type) material or metal‐semiconductor Schottky barriers. Practically in imaging, it can be achieved in field effect transistor (FET)‐like structure to significantly increase the amounts of carriers,^[^ ^165^ ^]^ giving a photocurrent driven by external voltage or by introducing local chemical doping. For those 2D materials owning semi‐metal properties like graphene, it will get a relatively large dark current with external voltage excitation. Moreover, the thermal mechanism is always unavoidable in the designed photodetectors. Thus, PTE will dominate in specific photodetectors especially observed for a nonuniformity doping. When PTE dominates, a photo‐excited e‐h pairs in graphene will lead to ultra‐fast (10–50 fs) heating of carriers, which causes the non‐uniform heat between 2D materials and semiconductor or metal.^[^ ^166^ ^]^ The light‐induced temperature increases the difference of work function between metal contact and 2D materials, generating a photo‐thermoelectric voltage (*V*_PTE_). Seebeck coefficient describes the magnitude of temperature affecting from Mott relation.^[^ ^167^ ^]^ With the different Seebeck coefficients in different materials, it will lead an unbalance in different types of material like graphene p–n junctions. When a symmetry design is introduced, the conductance of graphene can be changed when heated by the incident photons,^[^ ^168^ ^]^ which calls the PBE in photodetectors. When temperature keeps constant, the current in the photodetector is linear to the external bias as the conductance of graphene is constant. PBE photocurrent is generated by heating from the light illuminance. The number of carriers generated in conducting channel will contribute to PBE.

In reality, there is a main contributor in a specific photodetector even though there are hybrid photocurrent generation. Thus, a design should be carefully considered to choose the most suitable mechanisms, avoiding a contradictory photocurrent encountered.

### 5.2 High Speed Photodetectors with 2D Materials

Photodetectors play the role of receivers in communication systems, which can convert the optical signals to electrical signals. Germanium^[^ ^5^, ^169^, ^170^ ^]^ and III/V materials^[^ ^171^, ^172^, ^173^ ^]^ have been widely used in the integrated photodetectors in telecommunication PICs. However, the most important property of the photodetector is the bandwidth, which determines the base line of the data capacity. Since the first graphene‐based photodetector was reported in 2009,^[^ ^49^ ^]^ researchers have focused their attention on 2D materials to improve the performance of the photodetector. Using 2D materials in the optical communication systems can improve the speed significantly since the 2D materials have much higher mobility than traditional semiconductors.^[^ ^26^ ^]^ In addition, a high responsivity in photodetectors can increase the intensity of the detected signal and improve the quality of the receiver in an optical communication system. Direct‐incident type photodetectors, where the light illuminate from free space, have large photocurrent generation showing an outstanding photoresponsivity. Waveguide type photodetectors exhibit an ultra‐fast response time since a short electrical channel in there. In the communication systems, we need to integrate the photodetector into an electron/optical hybrid system. Therefore, it is better to place the photodetector and other optical/photon devices on the same plane. Thus, we will mainly focus on the waveguide type photodetectors in this overview, since the waveguide can be interconnected with other optical components such as MRR or MZI and can furthermore enhance the light‐matter interaction benefited from the waveguide length. We have compared the state‐of‐the‐art 2D material‐based photodetectors and composed a comprehensive overview about them.

#### 5.2.1 Graphene‐Based Photodetectors

**Figure 9:**
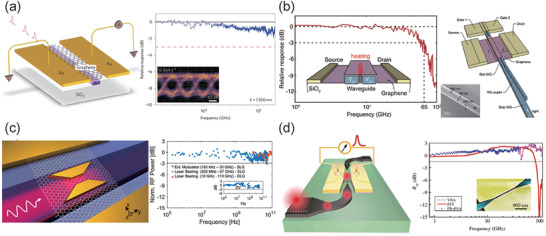
High speed graphene‐based photodetectors. a) The chip‐integrated ultrafast graphene photodetector with a silicon strip waveguide (left). The frequency response indicated that the 3 dB bandwidth is more than 20 GHz (right). b) A novel slot waveguide photodetector achieved a bandwidth of 65 GHz driven by PTE in graphene. c) Proposed bowtie‐shaped nanosized metallic structures integrated on a silicon‐based waveguide with an ultrafast speed. d) The ultracompact graphene plasmonic photodetector on pure Si platform exhibit an ultrawide bandwidth larger than 110 GHz. a) Reproduced with permission.^[^ ^50^ ^]^ Copyright 2013, Springer Nature. b) Reproduced with permission.^[^ ^176^ ^]^ Copyright 2016, American Chemical Society. c) Reproduced with permission.^[^ ^36^ ^]^ Copyright 2018, American Chemical Society. d) Reproduced under the terms of Creative Commons Attribution 4.0 (CC BY).^[^ ^35^ ^]^ Copyright 2020, The Authors, published by De Gruyter.

Demonstrated in 2009, a graphene photodetector with an ultrafast response was proposed by Xia et al.,^[^ ^49^ ^]^ which laid the foundation of the application of graphene in high‐speed optical communications. To allow graphene photodetectors to be interconnected with other optical devices, Gan et al. proposed a chip‐integrated ultrafast graphene photodetector in 2013,^[^ ^50^ ^]^ as shown in **Figure** **9**a. This photodetector used metal‐graphene‐metal asymmetric electrodes, where the symmetry of the internal electric‐field profile was broken leading to an apparent large photocurrent. A maximum photoresponsivity of 0.1 mA W^−1^ and response rates of 20 GHz were achieved at the wavelength of 1550 nm. The photocurrent of the structure is mostly generated by PVE in graphene, where the injected light separates electron–hole pairs in the lateral position of the graphene bands. This genius asymmetric design can generate two different band bending with opposing gradients occurring at the two electrode junctions. One year later, the bandwidth of this type of photodetector has been increased to 41^[^ ^174^ ^]^ and 42 GHz^[^ ^175^ ^]^ by modifying the media material between graphene and waveguide. Up to 2016, Schuler et al. proposed a novel slot waveguide photodetector and achieved a bandwidth of 65 GHz, where the PTE is dominated in this design, shown in Figure 9b.^[^ ^176^ ^]^ This photodetector used two gates for electrical and optical characterization, by which the Seebeck coefficient is controlled. Soon after, a graphene photodetector was successfully fabricated in a 6’’ wafer process line with a bandwidth over 76 GHz. The generated photocurrent is from the principle of PBE, where two symmetric electrodes is laid on both sides of a silicon waveguide without a doping gradient. The bolometric photo current is generated due to a temperature induced resistance variation of a graphene channel.^[^ ^168^ ^]^ Unlike the structures mentioned above, Echtermeyer et al. proposed that the photovoltage can be enhanced by a plasmonic structure in graphene, thus the efficiency of the photodetector can be increased by up to 20 times.^[^ ^177^ ^]^ Plasmonic waveguide photodetectors break through the limits of the waveguide‐integrated photodetector, whose mode overlap with graphene is rather restricted. Ma et al. proposed a bowtie‐shaped nanosized metallic structures integrated on a silicon‐based waveguide,^[^ ^36^ ^]^ which exhibit a photoresponse up to at least 110 GHz, shown in Figure 9c. The silicon waveguide is buried under a silicon nitride isolation thin layer, then graphene and two metal pads are stacked on top of the silicon waveguide. This special structure can excite the surface plasmon polaritons (SPPs) enhancing the external responsivity of the photodetector. Different from the bowtie structure, Ding et al. proposed an ultracompact graphene plasmonic photodetector on pure Si platform exhibiting an ultrawide bandwidth larger than 110 GHz,^[^ ^35^ ^]^ as shown in Figure 9d. Light is coupled into the silicon grating coupler and then goes through the taper tail of the silicon waveguide exciting the plasmonic mode in the metal slot waveguide. The two metal pads are Ti and Pd respectively, which can break the symmetry of the energy band of the graphene, leading to a higher responsivity. Moreover, this structure is easy to be integrated with other optical components since the graphene is at the same plane as the silicon waveguide. At the beginning of 2020, a graphene‐based plasmonic photodetector working in wavelength of 1.55 and 2 µm has been demonstrated, whose plasmonic mode is achieved by a narrow strip metal on top of the graphene.^[^ ^178^ ^]^ Although the bandwidth is relatively reduced by set‐up limit, it proposed a new type of structure which can be used in the mid‐IR field extended to 2 µm communications.

From the overview above, graphene photodetectors have been developed for ten years while the graphene is still a youth material in the field of research, and commercialization is just around the corner. However, graphene always has a relatively large dark current due to its zero bandgap. BP, which are synthesized as 2D materials later than graphene, have also emerged in recent years. Using its excellent and unique properties, a lot of photodetectors based on BP have been designed and demonstrated by directly induced light.^[^ ^179^, ^180^, ^181^, ^182^, ^183^, ^184^, ^185^ ^]^ However, the waveguide‐integrated photodetectors should be investigated as the same reasons as graphene photodetector.

#### 5.2.2 Other 2D Material‐Based Photodetectors

**Figure 10:**
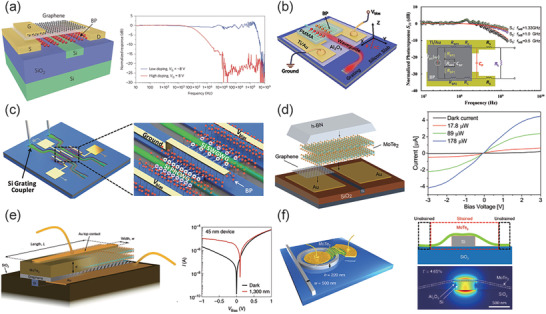
State‐of‐the‐art BP and TMDs photodetectors. a) 3D illustration of BP/graphene hybrid waveguide photodetector, featuring a few‐layer graphene top‐gate. b) BP‐based photodetector working in mid‐IR wavelength‐band. The frequency response with a 3 dB bandwidth of 1.33 GHz and equivalent circuit are shown in the right. c) Schematic of BP/PhCW hybrid photodetector, where the PhCW is utilized to enhance light‐matter interaction length. d) Schematic of a MoTe_2_ photodetector in the left, and the current in relation with bias at different power of light are shown in the right. e) Schematic of a waveguide‐integrated vertical MoTe_2_‐graphene van der Waals heterostructure photodetectors. The photocurrent is plotted in the right. f) Demonstrated MoTe_2_ photodetector utilized strain engineering of 2D film. a) Reproduced with permission.^[^ ^186^ ^]^ Copyright 2015, Springer Nature. b) Reproduced with permission.^[^ ^188^ ^]^ Copyright 2019, WILEY‐VCH GmbH. c) Reproduced with permission.^[^ ^34^ ^]^ Copyright 2020, WILEY‐VCH GmbH. d) Reproduced with permission.^[^ ^191^ ^]^ Copyright 2018, American Chemical Society. e) Reproduced with permission. Copyright 2020, Springer Nature.^[^ ^192^ ^]^ f) Reproduced with permission.^[^ ^31^ ^]^ Copyright 2020, Springer Nature.

In 2015, BP/graphene hybrid waveguide photodetector was demonstrated, as shown in **Figure** **10**a, which used the graphene top gate to electrostatically tune the carrier type and concentration in the BP layer, achieving a bandwidth exceeding 3 GHz.^[^ ^186^ ^]^ The photocurrent is dominated by the PVE, and the BP photodetector has high responsivity with low dark current. Since the BP thin film in this photodetector is exfoliated from a bulk material, it can be further improved by controlling the number of layers of BP to be tailored for a specific wavelength so that both the responsivity and dark current can be optimized. Benefit from the adjustable bandgap of BP, Huang et al. bring the BP into mid‐IR communication applications by designing a waveguide type photodetector, where the light can be coupled into the waveguide by a grating coupler.^[^ ^187^ ^]^ Recently, a BP‐based photodetector working in mid‐IR wavelength‐band of 2 µm is demonstrated with a 3 dB bandwidth of 1.33 GHz, as shown in Figure 10b.^[^ ^188^ ^]^ This structure used a PMMA upper‐cladding to protect BP from oxidization, and it experimentally pushed the BP into the 2 µm communication practice. To maintain high responsivity BP‐based photodetectors, a PhCW was utilized to enhance light‐matter interaction length, as shown in Figure 10c.^[^ ^34^ ^]^ With the help of PhCW, BP photodetectors can be realized in high‐performance on‐chip MIR systems and further utilized in widespread applications.

TMDs, such as MoS_2_,^[^ ^51^ ^]^ PtSe_2_,^[^ ^189^ ^]^ and WSe_2_^[^ ^190^ ^]^ et al., have also been achieved as a photodetector. Since the bandgap of most TMDs are located in the visible range, rare of them are suitable for telecommunications. Besides, they are mostly achieved as a free space photodetector but not in an integrated waveguide, where the responsivity rather than response speed is the goal they focused on. MoTe_2_ is special in TMD family with an infrared bandgap. In 2017, Bie et al. demonstrated a special optoelectronic device for silicon photonic integrated circuits based on a bilayer MoTe_2_ p–n junction, which can work as light‐emitting diode and photodetector.^[^ ^103^ ^]^ The peak response wavelength of the photocurrent is 1160 nm with responsivity of 4.8 mA W^−1^, and it can operate in a bandwidth above 200 MHz. Ma et al. replaced a gold contact pad by graphene in a MoTe_2_ photodetector operating in the O‐band of the near‐infrared optical telecommunication spectral range, as shown in Figure 10d.^[^ ^191^ ^]^ The density of state of graphene varies with the shifts of its Fermi level, which helps to efficiently extract photocarriers. A waveguide‐integrated vertical MoTe_2_‐graphene van der Waals heterostructure photodetectors was recently reported, which can work in the 1260–1360 nm range, as shown in Figure 10e.^[^ ^192^ ^]^ Thanks to the ultra‐short carrier drift path, the device can experimentally achieve the highest responsivity of 0.2 A W^−1^ with bandwidth over 30 GHz. The strain engineering in MoTe_2_ photodetector is demonstrated by Maiti et al. and achieved a responsivity of 0.5 A W^−1^ at 1550 nm over silicon MRR, where the MoTe_2_ is transferred on a silicon MRR keeping strained near waveguide region leading to an energy bandgap change from 1.0 eV to 0.8 eV, as shown in Figure 10f.^[^ ^31^ ^]^ This special design can bring TMDs into the 1550 nm telecommunication range without introducing additional materials. The method of using sub‐bandgap engineering of 2D layered TMDs to realize the photodetection of telecommunication band has been demonstrated on the substrate, including MoSe_2_/WSe_2_^[^ ^193^ ^]^ and PtS_2_/PtSe_2_^[^ ^194^ ^]^ heterojunctions, etc. It can be expected that utilizing mature transfer technology to integrate such heterojunctions on waveguides is a promising way for realizing on‐chip photodetection applications.

To pursue high performance optoelectronic devices based on 2D materials, van der Waals heterostructure of 2D materials is an important option but mostly achieved in direct‐incident type photodetectors. Heterostructures can combine the advantage of individual 2D material and realize unique electronic, optoelectronic properties and multifunctional applications. Duan et al. fabricated MoS_2_–MoSe_2_ and WS_2_–WSe_2_ lateral heterostructures through lateral epitaxial growth process.^[^ ^195^ ^]^ In sharp contrast to that of individual TMDs, for both heterostructures, the gain, carrier mobility, photoresponse speed were significantly enhanced. Then, by utilizing a two‐step direct epitaxial growth means, Sun et al. successfully fabricated the van der Waals heterostructures based on FETs.^[^ ^196^, ^197^ ^]^ The ON/OFF ratio, rectification ratio, and photoresponsivity were dramatically improved. Li et al. synthesized self‐aligned and scalable WSe_2_–MoS_2_ monolayer lateral heterojunction through two‐step and location‐selective chemical vapor deposition process.^[^ ^198^ ^]^ Moreover, the heterojunction exhibited type‐I band alignment behavior rather than commonly adopted type‐II alignment at the interface. To realize ultrasensitive phototransistor, Choi et al. fabricated a photodetector based on WSe_2_−MoS_2_ heterojunction.^[^ ^199^ ^]^ The specific detectivity, photoresponsivity of the device were measured to be 5 × 10^11^ Jones and 2700 A W^−1^, respectively. Which were high enough for ultrasensitive detection applications. Not only the performance of the device can be enhanced through employing van der Waals heterostructure, but also the carrier dynamics at the interface can be modified. Nonthermalized photocarrier energy was realized through employing a metal–insulator–semiconductor heterojunction.^[^ ^200^ ^]^ The upconversion photoluminescence range from NIR to visible band was achieved by photoinduced interlayer hole transfer from few‐layer graphene to WS_2_ followed by radiative recombination and exciton formation. These heterostructures are promising to be applied in future waveguide type photodetectors to achieve large photoresponsivity in on‐chip interconnections.

## 6 Summary and Perspectives

In this review, we introduced 2D materials in preparing and fabrication methods and summarized their applications in laser sources, modulators and photodetectors of the on‐chip platform. Hybrid integration with 2D materials can improve the performance of traditional optoelectronics. It is foreseeable to create an all‐integrated optoelectronic chip in a single platform. In all these experimental demonstrations, 2D materials exhibited extraordinary performance on integrated platforms. Thanks to the tunable bandgap, spin–valley correlation, and large exciton binding energy, 2D materials are promising for light emission device applications. Benefiting from the high carrier mobility, 2D material‐based modulators exhibit improved bandwidth and reduced energy consumption on integrated silicon platforms. In addition, photodetectors combined with 2D materials supplement the insufficient light‐matter interaction in pure silicon waveguide, resulting in a high responsivity and fast response time. Besides, graphene dominated the 2D material‐based optoelectronics since it has the highest carrier mobility and could be stably kept with a mature transferring/patterning technology. However, in some specific applications, BP and TMDs demonstrated a more outstanding performance than graphene. Since the BP and TMDs have a non‐zero bandgap, they are inherently suitable to work as the laser source and have lower dark current for photodetectors. Thus, we need to pick an optimum material in specific applications. With the advent of the fifth generation (5G) technology, the data capacity will grow exponentially. The energy costs need to be reduced while the speeds need to be increased. In next‐generation optoelectronic applications, artificial intelligence (AI) neuromorphic computing can significantly promote the performance of signal processing in telecommunications. We have seen several optical neural networks (ONNs) based on silicon substrate for on‐chip signal processing.^[^ ^201^, ^202^ ^]^ However, they can be further improved in size, energy consumption and bandwidth as long as the optical components replaced by 2D material‐based optoelectronics.

2D materials have been proven to be useful in telecommunications owing to their optoelectronic properties. The energy bandgap of graphene, BP and TMDs spans the range from MIR, NIR to visible light, responding the whole telecom band. Besides, since 2D materials are stacked by van der Waals forces, it is easy to transfer them on almost all kinds of surfaces. From the Section 2 we concluded, 2D materials can be prepared by liquid exfoliation and CVD wet transferring method, and furthermore to be patterned by O_2_ plasma etch in a skilled process. These fabrication methods bring them into future industrial applications. By cooperating with special optical structures, several novel perspectives are proposed to improve the behavior of 2D materials. For instance, the slow light effect in PhCW provides a longer interaction time, improving the modulation depth in modulators and responsivity in photodetectors. The resonance in MRR significantly increases the time of light‐matter interactions, where both absorption and heating effect are reinforced. The plasmonic structure excites the SPP mode on the 2D materials. Different from strip waveguide, the 2D materials serve as the waveguide part of the modulators and photodetectors, which forms a stronger electric field over the 2D materials. Besides, with a tiny footprint of plasmonic waveguide, the RC constant will be further reduced, bringing a large bandwidth in the devices. It is predictable that there will be more methods to improve the performance of 2D material‐based optoelectronics in the future. For now, the plasmonic waveguide achieved by metallic nanostructures is the most effective structure to boost the performance of modulators and photodetectors without large loss. The excited plasmonic wave in 2D materials provides a fully coverage in light mode and 2D materials. With an uncomplicated fabrication process, 2D materials enhanced by metallic nanostructures benefit from the promising advantages of high speed and low energy consumption. Thus, it is predicted that there will be large scale process of 2D materials in metallic nanostructures in the future.

On silicon integrated platforms, traditional PICs are difficult to integrate generation, modulation, and detection of light in an all‐in‐one silicon chip. 2D materials avoid the mismatch problem in the interface and promote the optical and electrical performance of the ultimate devices. So far, the demonstration of high speed photodetector can reach 110 GHz^[^ ^35^, ^36^ ^]^ but the 100 GHz bandwidth for modulators only stays in the simulation and design stage.^[^ ^116^ ^]^ Moreover, the insertion loss should be carefully considered for real applications especially in modulators. It is still a challenge to put the 100 GHz design into practice since it needs a more complicated fabrication process of double‐layer 2D materials. In addition, reducing the energy consumption further to the attoJoule level is essential for future large‐scale PICs.^[^ ^21^ ^]^ We can reduce the resistance of 2D materials by optimizing the preparing methods and increase the response of the signal by extremely enhancing light‐matter interaction in novel waveguide structures. The lack of efficient silicon‐based integrated light sources is often considered as the primary defect of monolithic integration on silicon platforms. Fortunately, through the hybrid integration with 2D materials, it is no longer difficult to integrate light sources on silicon platforms as they have been successfully demonstrated in integrated platforms, whose availability rekindled the possibility of future silicon‐based all‐in‐one platforms.

In summary, this review introduced the fundamentals of 2D materials ranging from fabrication to real applications. With the CVD and inkjet printing method, wafer‐scale process of chips with 2D materials can be foreseen. The 2D plasmonic devices with high speed and low energy consumption are promising to be applied in next‐generation optoelectronic interconnections. 2D materials are efficient to replenish the silicon integrated platforms for light sources, modulators and photodetectors, and will play an important role in achieving an all‐in‐one high‐speed silicon chip.

## Conflict of Interest

The authors declare no conflict of interest.
